# Fruit juice mediated multicomponent reaction for the synthesis of substituted isoxazoles and their in vitro bio-evaluation

**DOI:** 10.1038/s41598-021-03057-6

**Published:** 2021-12-07

**Authors:** Susheel Gulati, Rajvir Singh, Suman Sangwan

**Affiliations:** Department of Chemistry, Chaudhary Charan Singh Haryana Agricultural University, Hisar, 125004 India

**Keywords:** Environmental sciences, Chemistry

## Abstract

A simple, efficient and eco-friendly procedure for the synthesis of isoxazole derivatives **(4a–4h)** using one-pot three-component reaction between substituted aldehydes **(1a)**, methyl acetoacetate **(2a)** and hydroxylamine hydrochloride **(3a)** has been achieved in presence of *Cocos nucifera* L. juice, *Solanum lycopersicum* L. juice and *Citrus limetta* juice respectively. The homogeneity of synthesized compounds was confirmed by melting point and thin layer chromatography. The synthesized compounds were characterized by using ^1^H NMR, FTIR and CHN analyses and evaluated for in vitro herbicidal activity against *Raphanus sativus* L. (Radish seeds). The compounds **(4a–4h)** were also screened for their fungicidal activity against *Rhizoctonia solani* and *Colletotrichum gloeosporioides*. Antibacterial activity was also tested against *Erwinia carotovora* and *Xanthomonas citri*. From bio-evaluation data, it was found that compound **4b** was most active against *Raphanus sativus* L. (root) and *Raphanus sativus* L. (shoot) respectively. Compound **4b** was also found most active against both the fungus viz. *R. solani* and *C. gloeosporioides* showing maximum percentage growth inhibition i.e. 90.00 against *R. solani* and 82.45 against *C. gloeosporioides* at 2000 µg/mL concentration. Compound 4 h has shown maximum inhibition zone i.e. 3.00–9.60 mm against *Erwinia carotovora* at 2000 µg/mL concentration. Maximum *Xanthomonas citri* growth was also inhibited by compound 4 h showing inhibition zone 1.00–5.00 mm at highest concentration.

## Introduction

**Figure 1:**
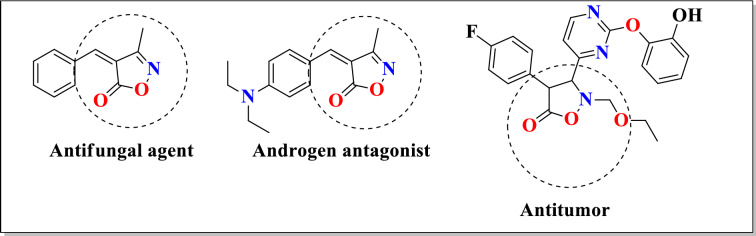
Some biologically important isoxazolone compounds.

Recently application of green chemistry for the formation of potential bioactive heterocyclic moiety has turned out the key area of research for organic chemist due to growing concern over environmental issues^1^. Therefore, the development of non-hazardous synthetic protocol gained the particular attention of synthetic chemist as frontier task in present scenario^2,3^. Lately multi-component reactions (MCRs) are important approach in organic chemistry in which a single operation is enough to form desired product from the well-defined condensation of three or more substrate molecules^4^. Therefore, multi-component reactions have emerged as a clean and facile route for synthesis of potential bioactive heterocyclic compounds and preferred over the stepwise synthetic methods. The beauty of multi-component reactions is eco-friendly nature, less time consumption, excellent atom economy, no need of column chromatography, minimum waste disposal etc^5^. Isoxazole derivatives are important class of heterocyclic compounds and they are more abundant in nature and show broad range of biological and pharmaceutical activities viz. *β*-adrenergic receptor antagonists, immunosuppressive, anti-inflammatory, antibacterial, HDAC inhibitors, antifungal, antitumor, antioxidant, antiprotozoal, antiviral, anti-tubercular, anti-HIV, analgesic and anti-androgens (II)^6–20^. Some of the biologically active isoxazole derivatives have shown in Fig. 1. The isoxazole ring system is found in a variety of naturally occurring compounds and biologically active molecules. They are principally useful in medicine viz. ibotenic acid (potent agonist), muscimol (potent GABA_A_ agonist, activating the receptor for the brain’s principal inhibitory neurotransmitter, GABA), isoxazole-4-carboxylic acid (anti-proliferative), valdecoxib (anti-inflammatory), leflunomide (immunosuppressive disease-modifying antirheumatic drug (DMARD)) and cloxacillin (*β*-lactam antibiotics). Thus, synthesis of isoxazole-containing compounds is of considerable interest. In the literature, it was found that *α,β*-unsaturated isoxazol-5(4*H*)-one derivatives have been prepared via one-pot three component condensation reaction of *β*-oxoesters, hydroxylamine hydrochloride and substituted aldehydes by use of catalytic amounts of sodium benzoate, sodium sulphide, sodium silicate, tartaric acid, pyridine, sodium ascorbate, sodium tetraborate and boric acid^21^. But these methods have some limitations such as harsh reaction conditions, expensive procedure, tedious work-up process, less product yield, less atom economy and completion of reaction time was also more. Based on these findings and our on-going efforts towards synthesis of isoxazole derivatives, in this paper we reported one-pot three components clean and facile synthesis of isoxazole derivatives in presence of fruit juices viz. *Cocos nucifera* L. juice, *Solanum lycopersicum* L. juice and *Citrus limetta* juice. Moderate to excellent product yield, less reaction time, reduce use of strong acids and bases, high atom economy are some merits of present methodology.

## Results and discussion

**Scheme 1:**
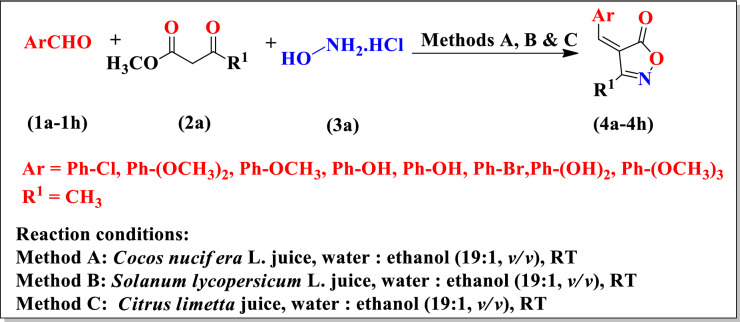
Synthesis of substituted isoxazole derivatives **(4a–4h).**

Initially, for optimization the reaction conditions hydroxylamine hydrochloride (20 mmol), methyl acetoacetate (20 mmol) was stirred in 20 mL water: ethanol (19:1, *v/v*) at room temperature for 15 min and then 4-Hydroxy-3-methoxybenzaldehyde was added to reaction mixture (Scheme 1).

**Figure 2:**
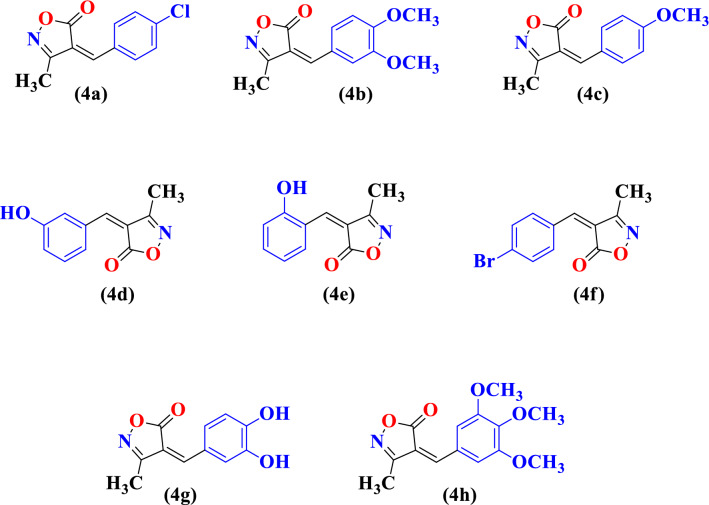
Substituted isoxazole derivatives **(4a–4h)**.

First, we performed model reaction in presence of *Cocos nucifera* L. juice at room temperature. We found that when the amount of *Cocos nucifera* L. juice was only 4.0 mL in reaction mixture then yield of reaction was less (78%) and completion of reaction time was also more (Table 1, Entry 1). Than we increased the amount of *Cocos nucifera* L. i.e. 6.0 mL, 8.0 mL and 10.0 mL respectively and we observed maximum yield of product (92%) and reaction time was also less (Table1, Entry 4). After having these encouraging results, next we explored the same model reaction in presence of *Solanum lycopersicum* L. juice and *Citrus limetta* juice. We observed that maximum yield (90%) (Table 1, Entry 4), and (95%) (Table 1, Entry 4) was obtained in presence of *Solanum lycopersicum* L. juice (10.0 mL) and *Citrus limetta* juice (10.0 mL) respectively. Under these optimized conditions, we further explored the substrate scope of this methodology using variety substituted benzaldehydes viz. 4-chlorobenzaldehyde **(1a)**, 3,4-dimethoxybenzaldehyde **(1b)**, 4-methoxybenzaldehyde **(1c)**, 3-hydroxybenzaldehyde **(1d)**, 2-hydroxybenzaldehyde **(1e)**, 4-bromobenzaldehyde **(1f)**, 3,4-dihydroxybenzaldehyde **(1g)**, 3,4,5-trimethoxybenzaldehyde **(1h)**. The physical data of this study were presented in Table 2. All the synthesized compounds **(4a–4h)** were characterized by ^I^H NMR, FTIR and CHN analyses and shown in Fig. 2. From spectral study it was observed that the ^1^H NMR spectrum of compound viz. (*Z*)-4-(4-methoxybenzylidene)-3-methylisoxazol-5(4*H*)-one **(4c)** in DMSO-*d*_*6*_, exhibited a singlet at 2.25 ppm integrating for three protons of methyl group, singlet at 3.89 ppm integrating for three protons of aryl methoxy group, singlet at 7.74 ppm integrating for one proton of =CH group, multiplet at 7.05–8.50 ppm integrating for protons of aryl group. The compound **(4c)** also displayed IR absorptions at 1729, 1619, 1591, 1431 and 1276 cm^−1^ showed the presence of C=O, C=C aromatic, C=N, N–O and OCH_3_ respectively. The ^1^H NMR spectrum of compound viz. (*Z*)-4-(2-hydroxybenzylidene)-3-methylisoxazol-5(4*H*)-one **(4e)** in DMSO-*d*_*6*_, exhibited a singlet at 2.25 ppm integrating for three protons of methyl group, singlet at 10.85 ppm integrating for one proton of OH group, singlet at 8.20 ppm integrating for one proton of =CH group, multiplet at 6.87–8.77 ppm integrating for protons of aryl group. The ^1^H NMR spectrum of compound viz. (*Z*)-4-(4-bromobenzylidene)-3-methylisoxazol-5(4*H*)-one **(4f)** in CDCl_3_, exhibited a singlet at 2.29 ppm integrating for three protons of methyl group, singlet at 7.37 ppm integrating for one proton of =CH group, multiplet at 7.59–8.22 ppm integrating for protons of aryl group. In order to show the beauty of current protocol, the previous protocols and their yields for the synthesis were summarized in Table 3. We observed that *Cocos nucifera* L. juice, *Solanum lycopersicum* L. juice and *Citrus limetta* juice catalyst gives the best catalytic activity in terms of product yield and reaction time as compared to other catalysts in literature. Therefore the present procedure for synthesis of isoxazole derivatives is considered as sustainable and eco-friendly protocol.

**Table 1:** Model reaction of 4-Hydroxy-3-methoxybenzaldehyde (20 mmol), methyl acetoacetate (20 mmol) and hydroxylamine hydrochloride (20 mmol) using *Cocos nucifera* L. juice, *Solanum lycopersicum* L. juice and *Citrus limetta* juice as catalyst.

Entry	Catalyst concentration (mL)	Method A		Method B		Method C	
		Time (h)	Yield (%)	Time (h)	Yield (%)	Time (h)	Yield (%)
1	4.0	15	78	13	78	11	85
2	6.0	13	84	9	85	6	90
3	8.0	11	90	6	88	5	92
4	10.0	7	92	4	90	4	95

**Table 2:** Physical data of substituted isoxazole derivatives **(4a–4h)**.

Entry	Products	Ar	R^1^	Method A		Method B		Method C		mp (°C)
				Time (h)	Yield (%)	Time (h)	Yield (%)	Time (h)	Yield (%)	
1	**4a**	Ph-Cl	CH_3_	10	80	7	76	8	89	185–187
2	**4b**	Ph-(OCH_3_)_2_	CH_3_	8	81	6	89	10	90	210–212, (lit.^22^, 210–211)
3	**4c**	Ph-OCH_3_	CH_3_	5	92	8	90	8	92	172–174, (lit.^22^, 173–175)
4	**4d**	Ph-OH	CH_3_	6	90	7	92	8	83	200–202, (lit.^23^, 200–201)
5	**4e**	Ph-OH	CH_3_	8	85	5	80	4	80	198–200, (lit.^23^, 200–201)
6	**4f**	Ph-Br	CH_3_	7	80	6	78	8	76	178–180
7	**4g**	Ph-(OH)_2_	CH_3_	5	78	4	94	10	81	210–211, (lit.^22^, 212–214)
8	**4h**	Ph-(OCH_3_)_3_	CH_3_	6.5	82	4	91	6	82	194–196

**Table 3:** Comparison for different catalysts used for synthesis of isoxazole derivatives **(4a–4h)**.

S. no.	Catalyst	Solvent	Temperature (°C)	Time (min)	Yield (%)	References
1	Sodium benzoate (15 mol%)	Water	RT	60	85	^24^
2	Saccharose (20 mol%)	Solvent-free	100 °C	10	75	^25^
3	Cetyltrimethylammonium chloride (30 mol%)	Water	90 °C	240	89	^26^
4	Nano-ZnO (5 mol%)	Water	70 °C	60	94	^27^
5	Nano-CuI (1.2 mol%)	Water	Reflux	40	90	^28^
6	TBABr (10 mol %)	Water	Reflux	15	90	^29^
7	*γ*-Alumina (30 mol%)	Water	Reflux	50	80	^30^
8	*β*-Cyclodextrin (10 mol%)	Water–ethanol (9:1, *v/v*)	80 °C	15	92	^31^
9	Urea (10 mol%)	Water–ethanol (1:1, *v/v*)	RT	480	86	^32^
10	DABCO (5 mol%)	Water	Reflux	15	92	^33^
11	DCDBTSD (10 mol%)	Water	80 °C	60	85	^34^
12	[*Bmim*]OH (20 mol%)	Solvent-free	50–60 °C	45	90	^35^
**13**	***Cocos nucifera* L. juice**	Water : ethanol (19:1, *v/v*)	**RT**	**240**	**90**	Present work
**14**	***Solanum lycopersicum* L. juice**	Water : ethanol (19:1, *v/v*)	**RT**	**420**	**92**	Present work
**15**	***Citrus limetta* juice**	Water : ethanol (19:1, *v/v*)	**RT**	**240**	**95**	Present work

Significant values are in bold.

**Scheme 2:**
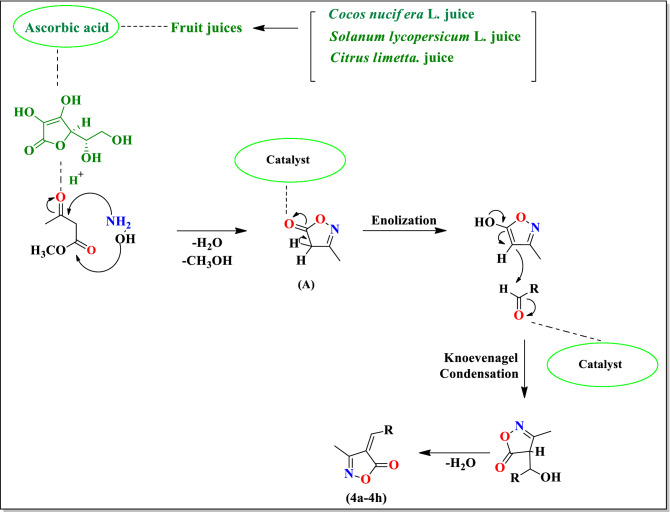
The possible mechanism for synthesis of substituted isoxazoles (**4a–4h**).

The possible mechanism for the formation of substituted isoxazole derivatives is shown in Scheme 2. According to this mechanism first of all there is formation of cyclized adduct **(A)** by the nucleophilic attack of the amino group and hydroxyl group of hydroxylamine hydrochloride to the carbonyl carbon of methyl acetoacetate in presence of *Cocos nucifera* L. juice, *Solanum lycopersicum* L. juice and *Citrus limetta* juice. The aldehyde was attacked on the cyclized adduct **(A)** and subsequent Knoevenagel adduct **(4a–4h)** is formed via removal of the water molecule.

### Herbicidal activity

**Figure 3:**
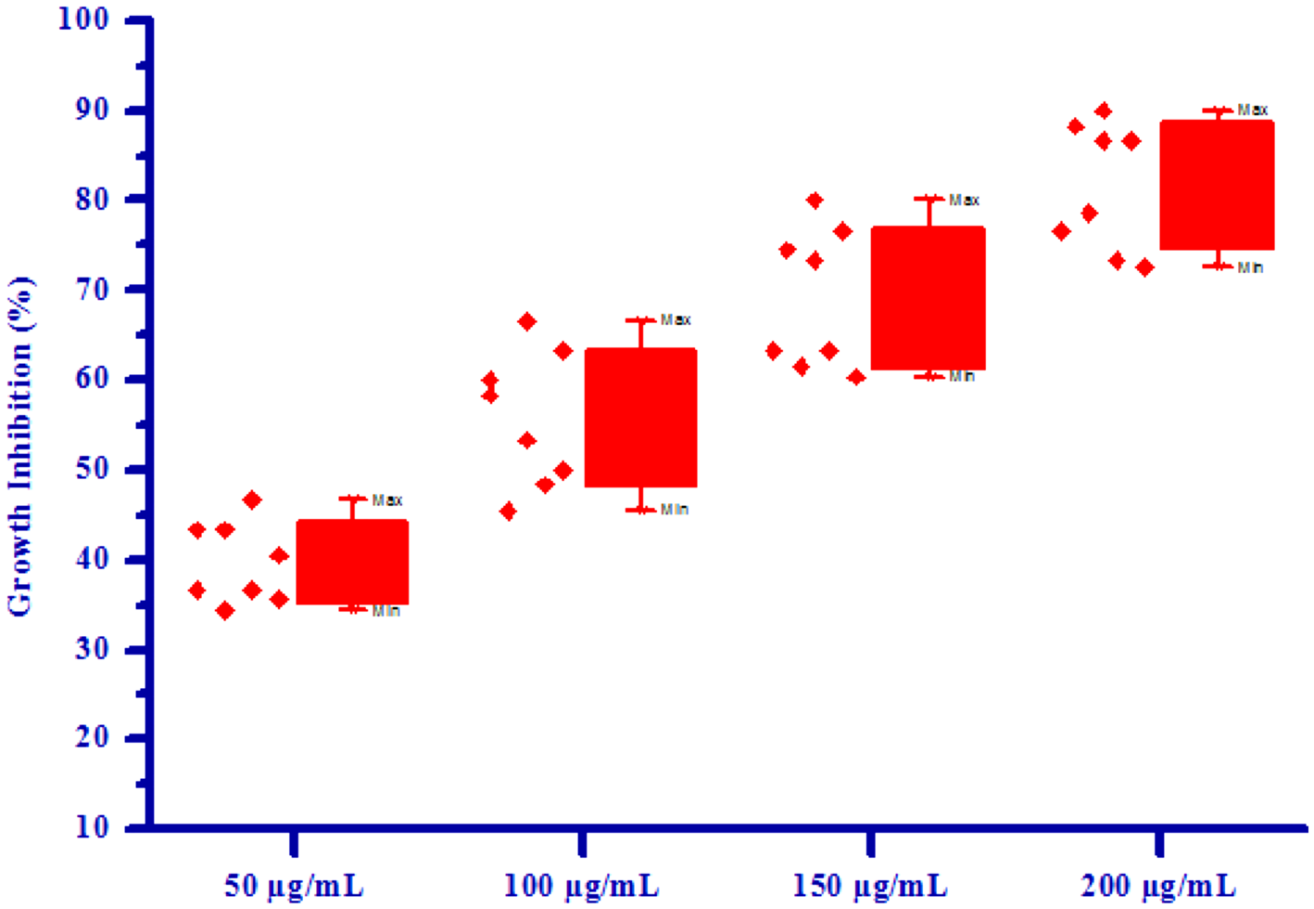
Box plot of substituted isoxazoles **(4a–4 h)** against *Raphanus sativus* L*.* (root).

**Figure 4:**
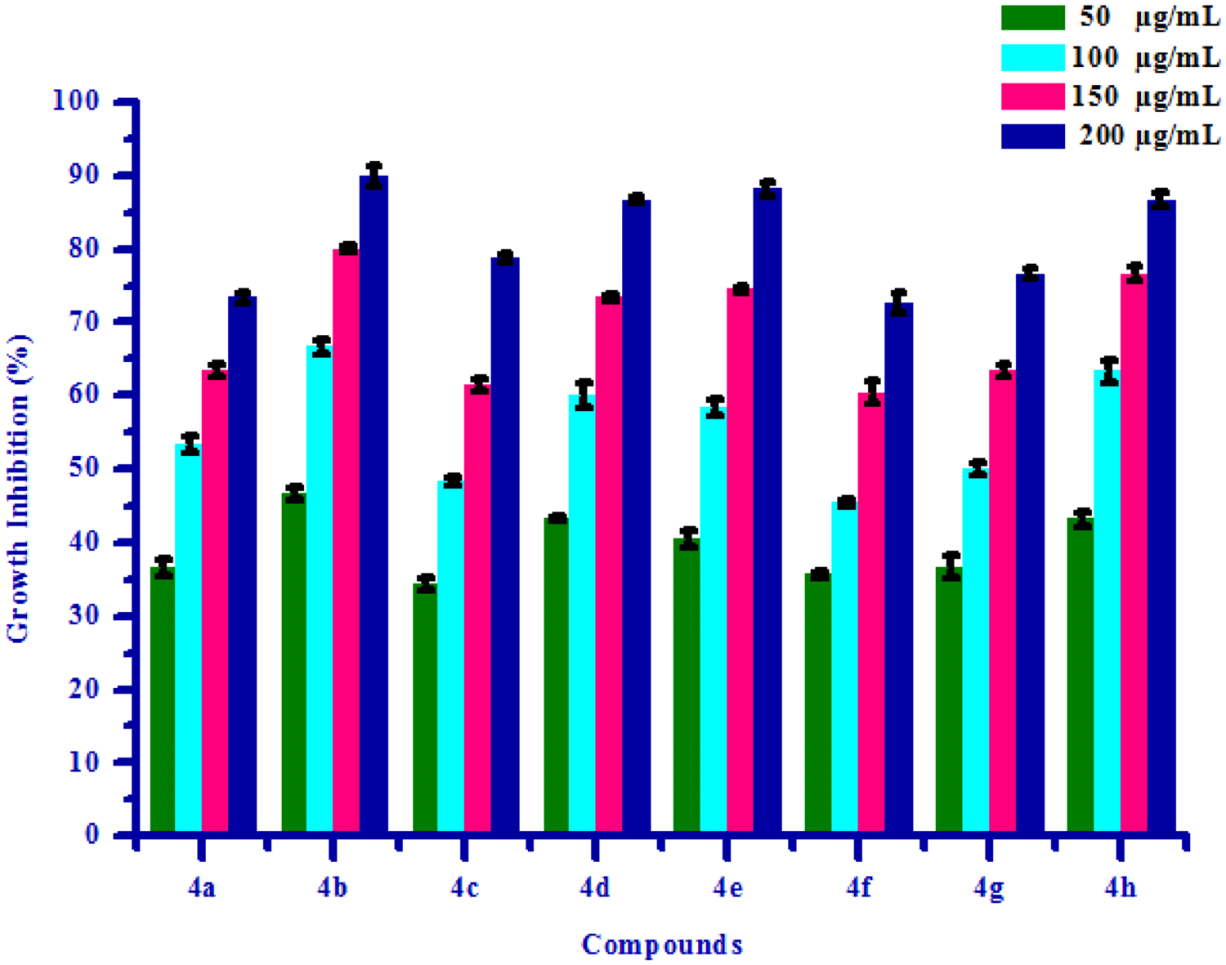
Herbicidal activity of substituted isoxazoles **(4a–4 h)** against *Raphanus sativus* L*.* (root).

**Figure 5:**
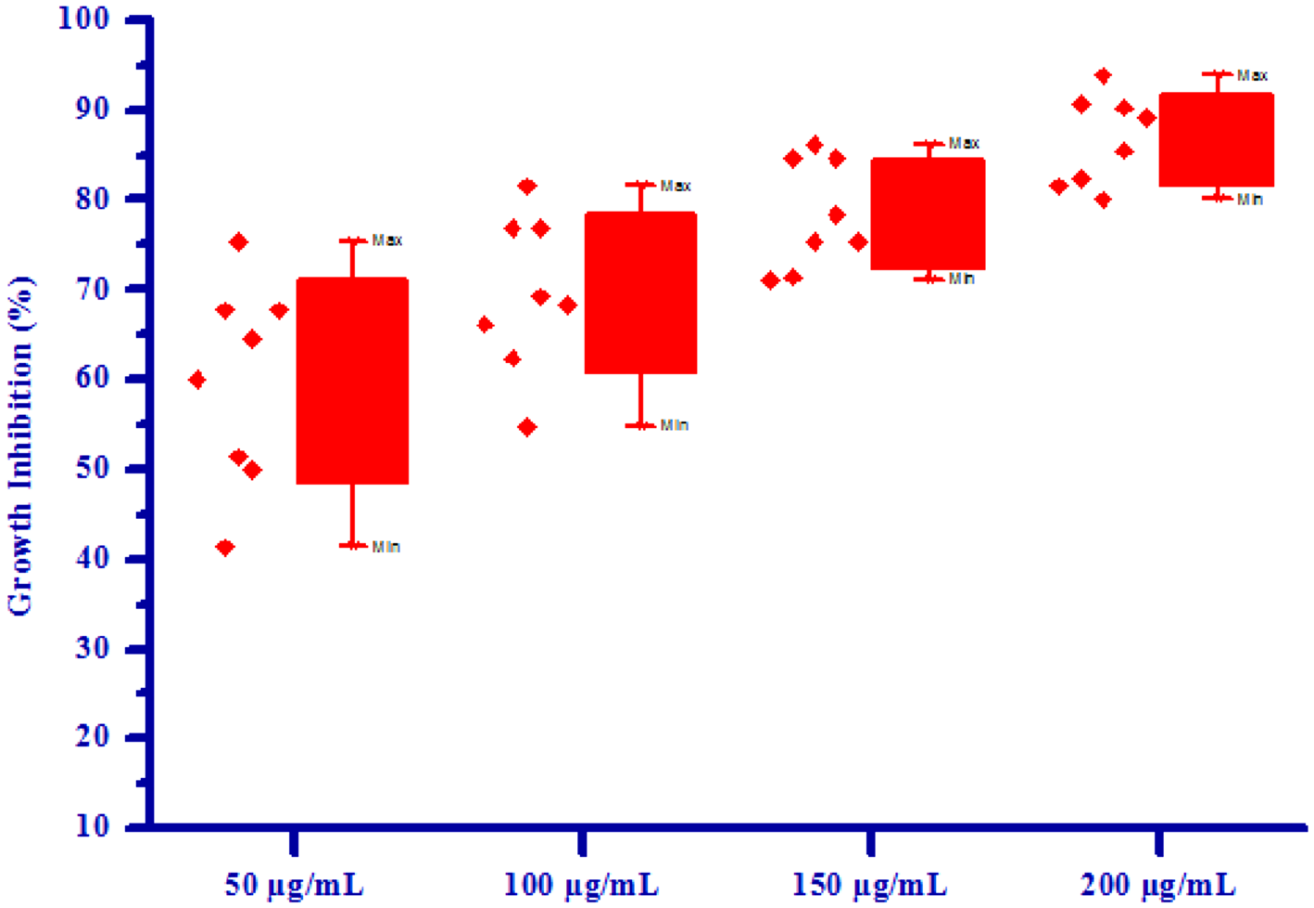
Box plot of substituted isoxazoles **(4a-4 h)** against *Raphanus sativus* L*.* (shoot).

**Figure 6:**
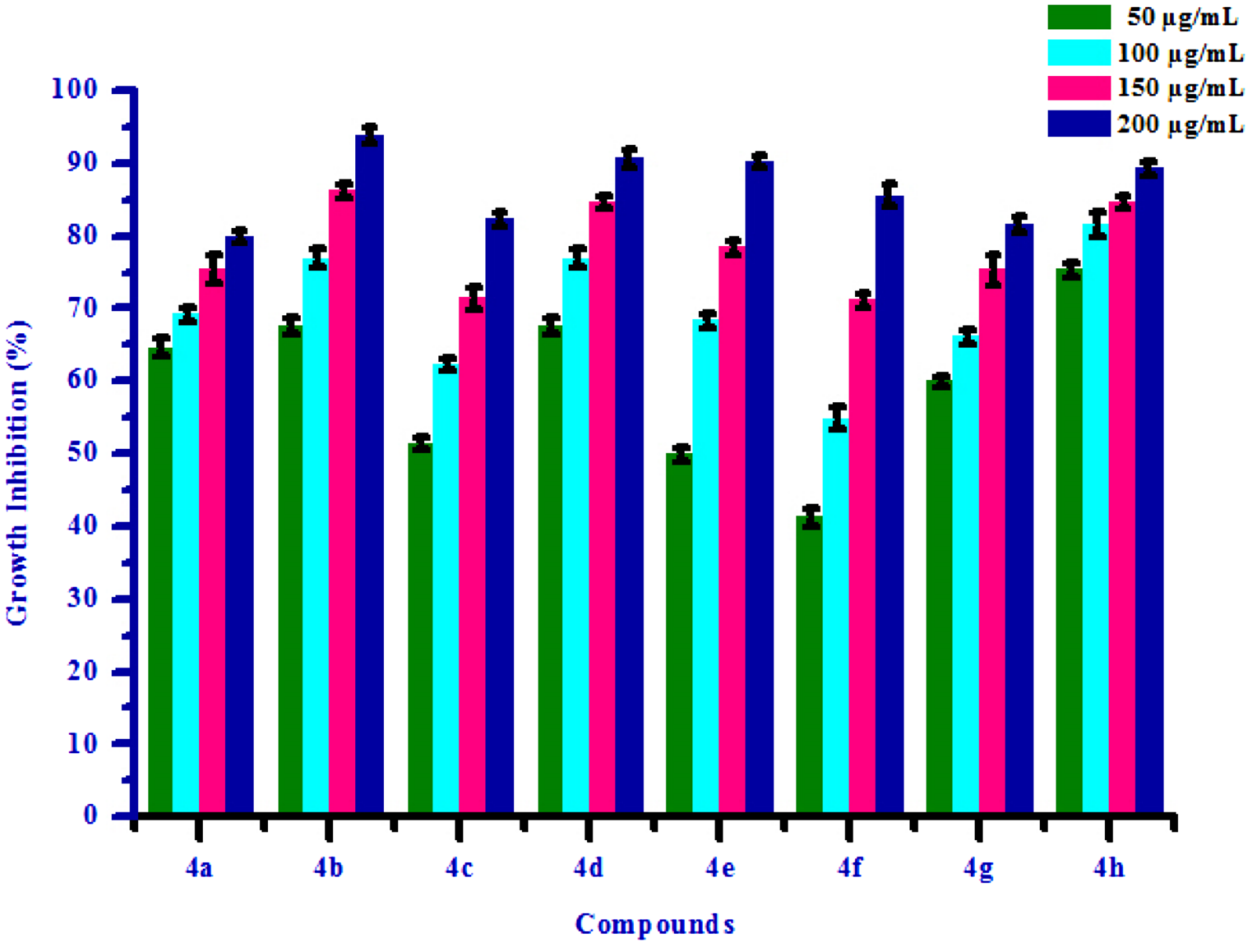
Herbicidal activity of substituted isoxazoles **(4a-4 h)** against *Raphanus sativus* L*.* (shoot).

All synthesized compounds **(4a–4h)** were screened for herbicidal activity against *Raphanus sativus* L. at various concentration 200, 150, 100 and 50 µg/mL as shown in Table 4. Synthesised compounds were diluted to 1000 µg/mL concentration as a stock solution. Herbicidal activity of synthesized compounds was evaluated against *Raphanus sativus* L. by inhibitory effect of the compounds on the growth of weed roots and shoots. The percentage of inhibition of growth was calculated from the mean differences between treated and control. From the herbicidal activity results, we observed that compound (*Z*)-4-(3,4-Dimethoxybenzylidene)-3-methylisoxazol-5(4*H*)-one **(4b)** was exhibited maximum percentage growth inhibition i.e. 90.00 against *Raphanus sativus* L. (root) and also exhibited maximum percentage growth inhibition i.e. 86.15 against *Raphanus sativus* L. (shoot) respectively at 200 µg/mL concentration. The growth inhibition may be attributed to substitution of methoxy group on phenyl ring. The box plot and graphical representation of herbicidal activity of all synthesized compounds **(4a–4h)** against *Raphanus sativus* L. seeds were shown in Figs. 3, 4, 5 and 6.

**Table 4:** Herbicidal activity of substituted isoxazoles **(4a–4h)**.

Compounds	Growth inhibition (%)							
	Root				Shoot			
	50 (µg/mL)	100 (µg/mL)	150 (µg/mL)	200 (µg/mL)	50 (µg/mL)	100 (µg/mL)	150 (µg/mL)	200 (µg/mL)
**4a**	36.66 ± 1.14	53.33 ± 1.07	63.33 ± 0.87	73.33 ± 0.67	64.61 ± 1.21	69.23 ± 0.93	75.38 ± 1.95	80.00 ± 0.83
**4b**	46.66 ± 0.93	66.60 ± 0.90	80.00 ± 0.50	90.00 ± 1.46	67.69 ± 1.11	76.92 ± 1.27	86.15 ± 0.89	93.84 ± 1.18
**4c**	34.36 ± 0.72	48.36 ± 0.49	61.45 ± 0.75	78.65 ± 0.55	51.39 ± 0.80	62.36 ± 0.78	71.45 ± 1.48	82.36 ± 0.99
**4d**	43.33 ± 0.40	60.00 ± 1.75	73.33 ± 0.40	86.66 ± 0.50	67.69 ± 1.12	76.92 ± 1.27	84.61 ± 0.94	90.76 ± 1.23
**4e**	40.52 ± 1.06	58.37 ± 0.99	74.50 ± 0.50	88.24 ± 1.00	49.92 ± 1.05	68.28 ± 0.97	78.36 ± 0.99	90.25 ± 0.94
**4f**	35.69 ± 0.43	45.48 ± 0.44	60.36 ± 1.52	72.58 ± 1.32	41.30 ± 1.24	54.78 ± 1.52	71.13 ± 1.01	85.48 ± 1.51
**4g**	36.66 ± 1.57	50.00 ± 0.82	63.33 ± 0.68	76.66 ± 0.82	60.00 ± 0.66	66.15 ± 1.00	75.38 ± 2.09	81.53 ± 1.14
**4h**	43.33 ± 0.98	63.33 ± 1.47	76.66 ± 0.97	86.66 ± 0.92	75.38 ± 0.95	81.53 ± 1.56	84.61 ± 1.10	89.23 ± 0.94

All values are mean ± S.D.

### Antifungal activity

**Figure 7:**
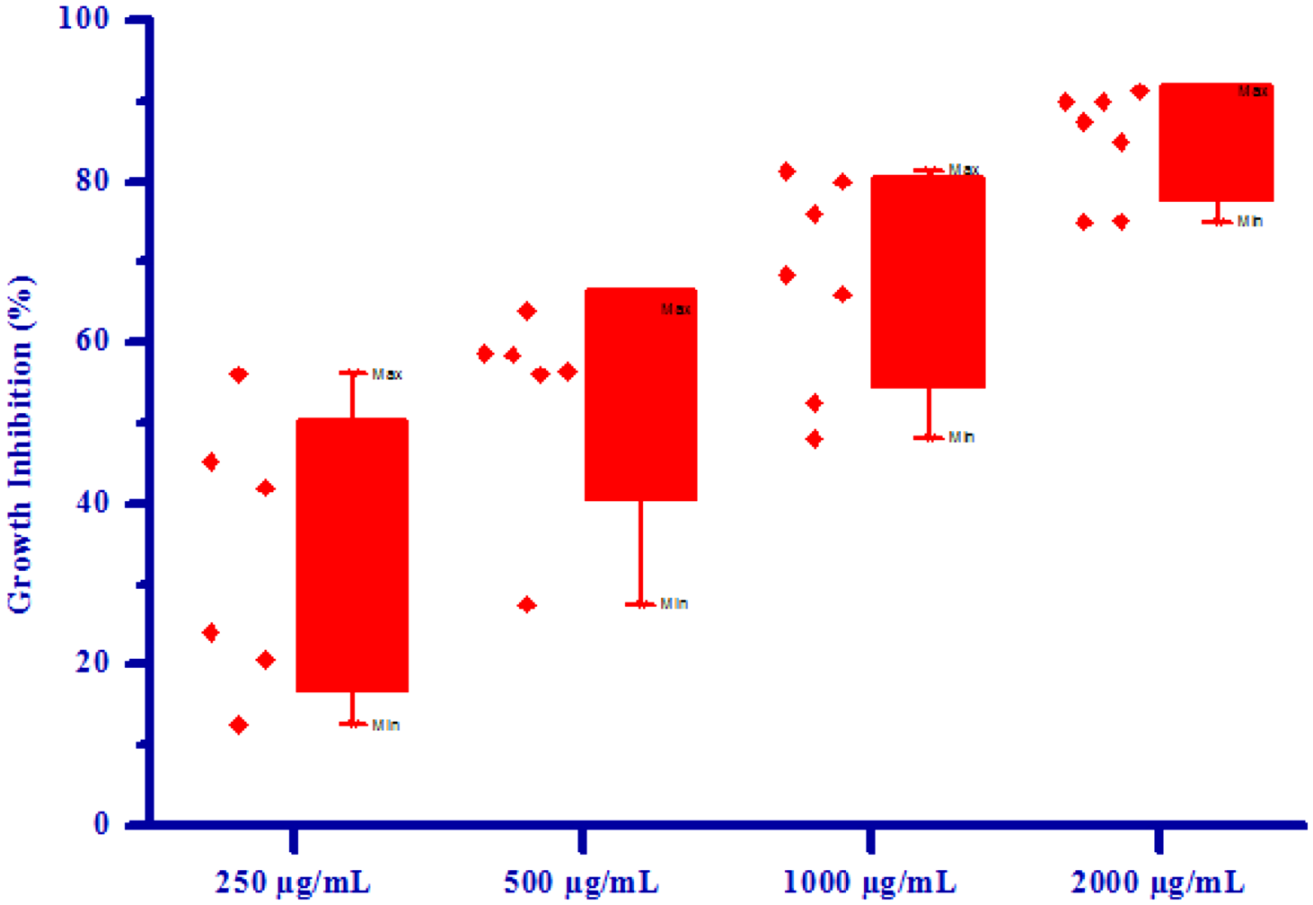
Box plot of substituted isoxazoles **(4a–4g)** against *Rhizoctonia solani*.

**Figure 8:**
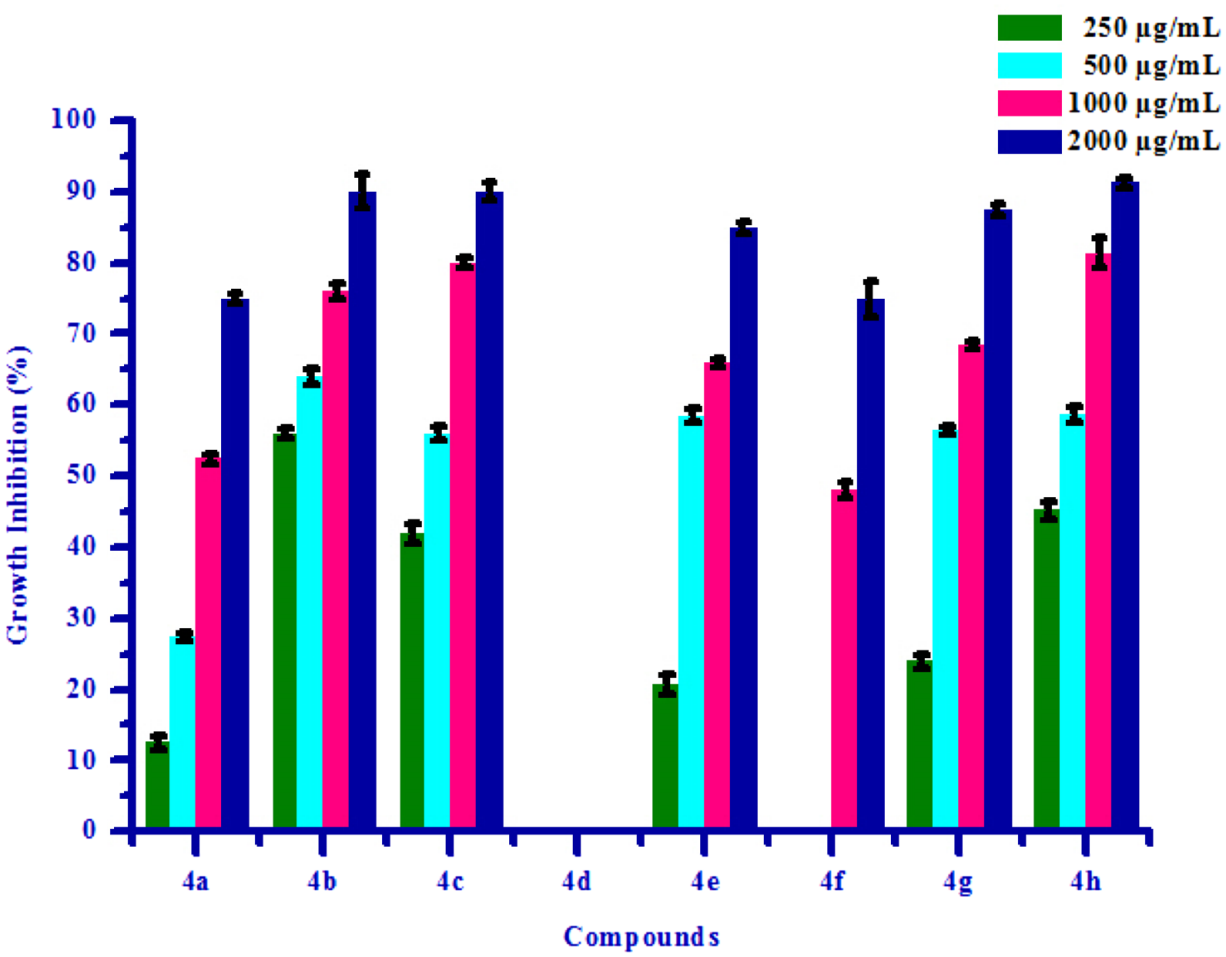
Antifungal activity of substituted isoxazoles **(4a–4h)** against *Rhizoctonia solani*.

**Figure 9:**
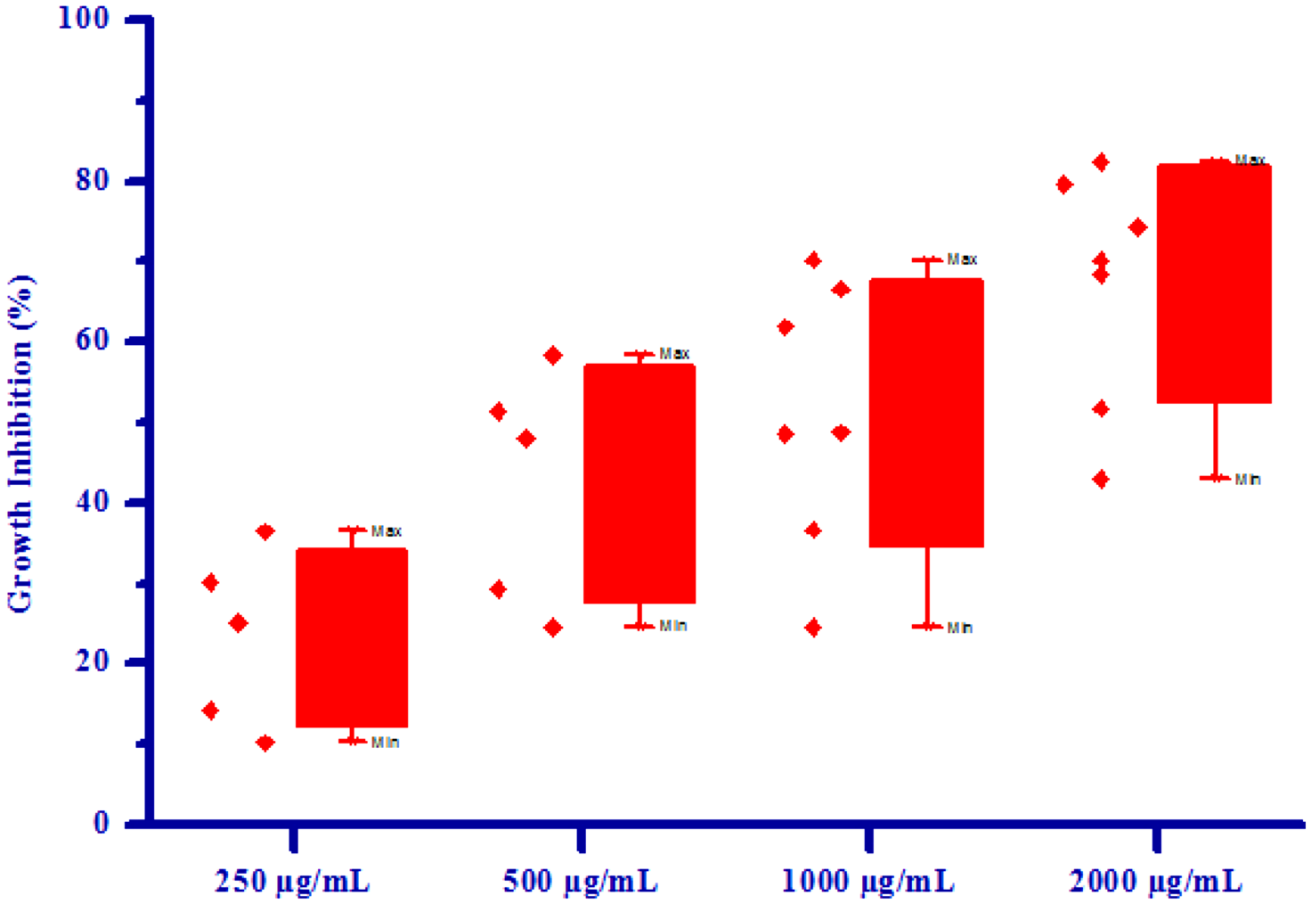
Box plot of substituted isoxazoles **(4a–4h)** against *Colletotrichum gloeosporioides*.

**Figure 10:**
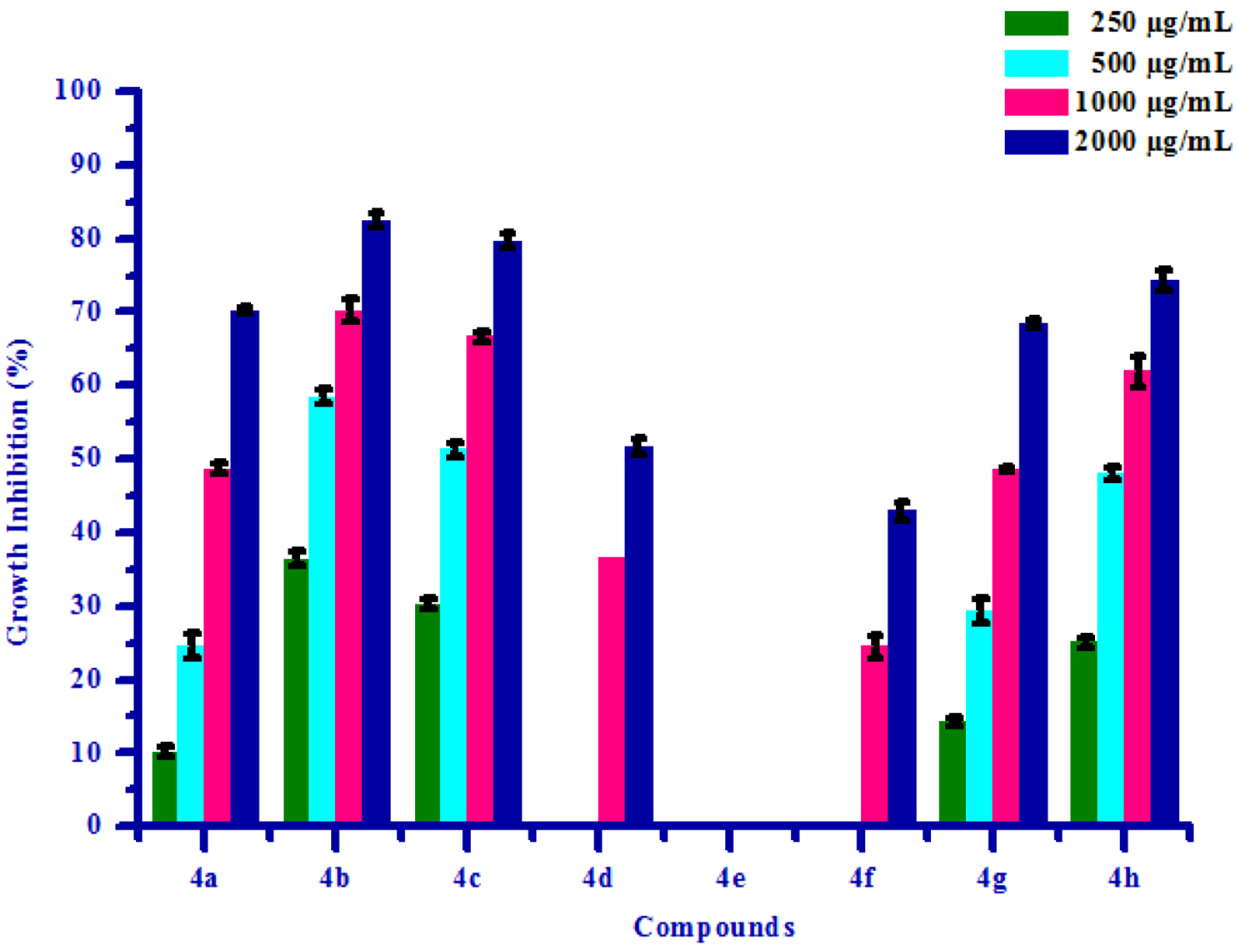
Antifungal activity of substituted isoxazoles **(4a–4h)** against *Colletotrichum gloeosporioides*.

All synthesized compounds **(4a–4h)** were screened for their fungicidal activity against 2 fungal strains viz. *Rhizoctonia solani* and *Colletotrichum gloeosporioides* by poisoned food technique method. DMSO was used as negative control against fungal strains. The result of antifungal activity of tested compounds is shown in Table 5. Most of synthesized compounds possess moderate to good activity against *R. solani* and *C. gloeosporioides* respectively. Compound **4d** showed no antifungal activity at all concentrations against *R. solani*, may be due to electron releasing nature of –OH group. Compound **4f** has shown no growth inhibition upto 500 µg/mL concentrations against *R. solani*. Compound **4f** exhibited 48.12 and 74.96% growth inhibition against *R. solani* fungus at 1000 µg/mL and 2000 µg/mL concentrations respectively, due to electron withdrawing effect of bromine substitution on phenyl ring. Compounds **4a, 4b, 4c, 4e, 4g** and **4h** exhibited 12.50, 27.50, 52.50, 75.00%, 56.00, 64.00, 76.00, 90.00%, 42.00, 56.00, 80.00, 90.00%, 20.75, 58.49, 66.03, 84.90%, 23.98, 56.42, 68.45, 87.36% and 45.23, 58.69, 81.35, 91.23% growth inhibition against *R. solani* fungus at 250 µg/mL, 500 µg/mL, 1000 µg/mL and 2000 µg/mL concentrations respectively, mainly due to presence of chlorine and methoxy substituion on phenyl ring. Compound **4e** showed no antifungal activity at all concentrations against *C. gloeosporioides*, may be due to electron releasing nature of –OH group. Compounds **4d** and **4f** has shown no growth inhibition at lower concentrations. Compound **4d** and **4f** exhibited 36.68, 51.78% and 24.56, 42.98% growth inhibition against *C. gloeosporioides* fungus at 1000 µg/mL and 2000 µg/mL concentrations respectively. Compounds **4a, 4b, 4c, 4g** and **4h** exhibited 10.23, 24.62, 48.75, 70.23%, 36.45, 58.45, 70.23, 82.45%, 30.25, 51.36, 66.60, 79.65%, 14.25, 29.35, 48.68, 68.45% and 25.12, 47.98, 61.89, 74.30% growth inhibition against *C. gloeosporioides* fungus at 250 µg/mL, 500 µg/mL, 1000 µg/mL and 2000 µg/mL concentrations respectively, mainly due to presence of chlorine and methoxy substituion on phenyl ring. From the fungicidal activity results, we concluded that compound **4b** was most likely against both the fungus viz. *R. solani* and *C. gloeosporioides* respectively. This result may be due to presence of methoxy group on phenyl ring. The box plot and graphical representation of antifungal activity of all synthesized compounds **(4a–4h)** against *Rhizoctonia solani* and *Colletotrichum gloeosporioides* were shown in Figs. 7, 8, 9 and 10.

**Table 5:** Antifungal activity of substituted isoxazoles **(4a–4h)**.

Compounds	Growth inhibition (%)							
	Fungi							
	*Rhizoctonia solani* (conc.) µg/mL				*Colletotrichum gloeosporioides* (conc.) µg/mL			
	250	500	1000	2000	250	500	1000	2000
**4a**	12.50 ± 1.07	27.50 ± 0.59	52.50 ± 0.71	75.00 ± 0.72	10.23 ± 0.68	24.62 ± 1.58	48.75 ± 0.69	70.23 ± 0.43
**4b**	56.00 ± 0.82	64.00 ± 1.06	76.00 ± 1.17	90.00 ± 2.35	36.45 ± 0.86	58.45 ± 0.95	70.23 ± 1.51	82.45 ± 1.04
**4c**	42.00 ± 1.37	56.00 ± 0.87	80.00 ± 0.66	90.00 ± 1.26	30.25 ± 0.73	51.36 ± 1.01	66.60 ± 0.67	79.65 ± 0.95
**4d**	a	a	a	a	a	a	36.68 ± 0.62	51.78 ± 1.09
**4e**	20.75 ± 1.44	58.49 ± 1.06	66.03 ± 0.56	84.90 ± 0.77	a	a	a	a
**4f**	a	a	48.12 ± 1.12	74.96 ± 2.50	a	a	24.56 ± 1.58	42.98 ± 1.30
**4g**	23.98 ± 0.93	56.42 ± 0.54	68.45 ± 0.69	87.36 ± 0.75	14.25 ± 0.52	29.35 ± 1.55	48.68 ± 0.35	68.45 ± 0.52
**4h**	45.23 ± 1.29	58.69 ± 1.01	81.35 ± 2.09	91.23 ± 0.72	25.12 ± 0.75	47.98 ± 0.87	61.89 ± 2.19	74.30 ± 1.44

All values are mean ± S.D.

a: no growth inhibition.

### Antibacterial activity

**Figure 11:**
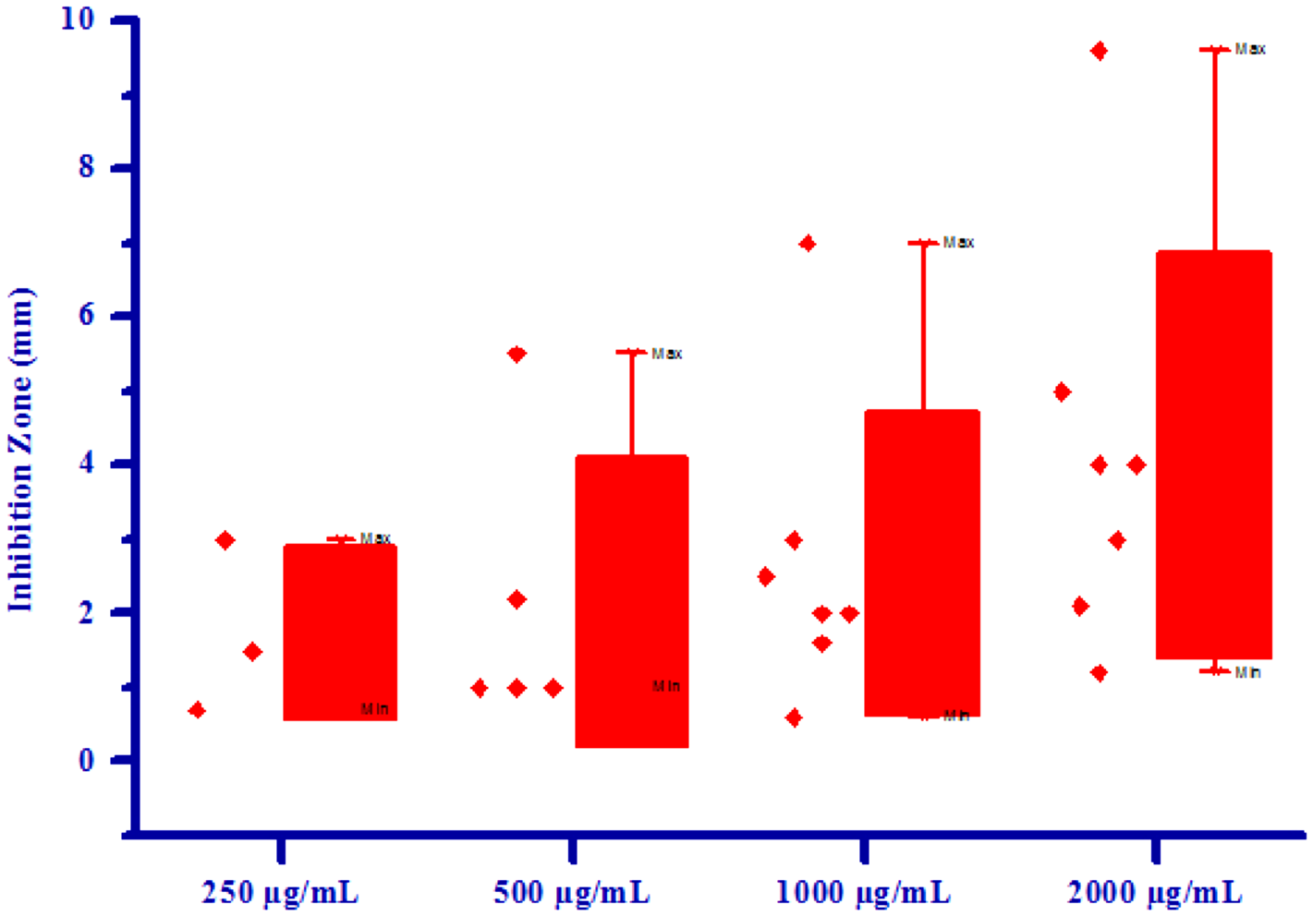
Box plot of substituted isoxazoles **(4a–4h)** against *Erwinia carotovora*.

**Figure 12:**
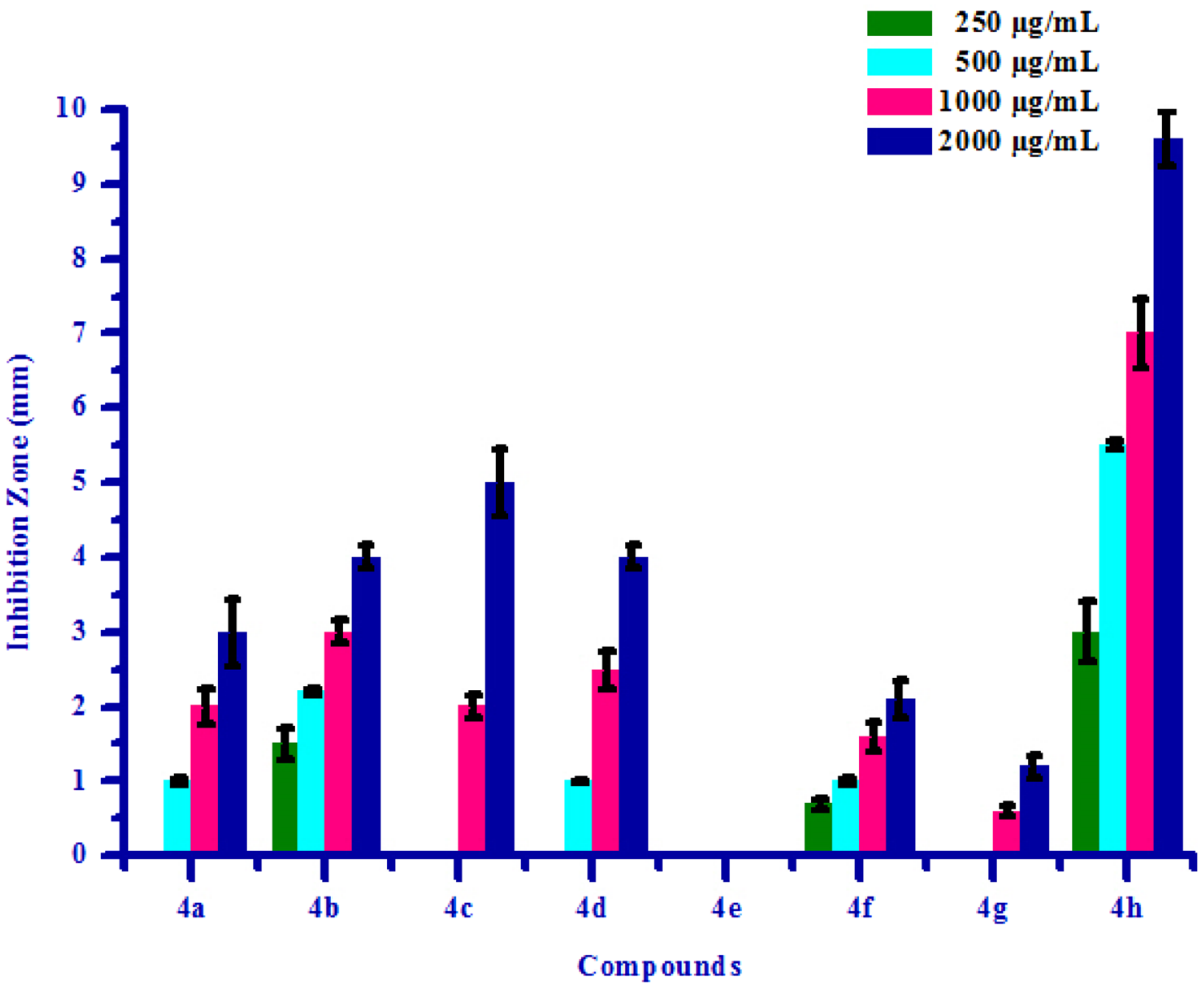
Antibacterial activity of substituted isoxazoles **(4a–4h)** against *Erwina carotovora*.

**Figure 13:**
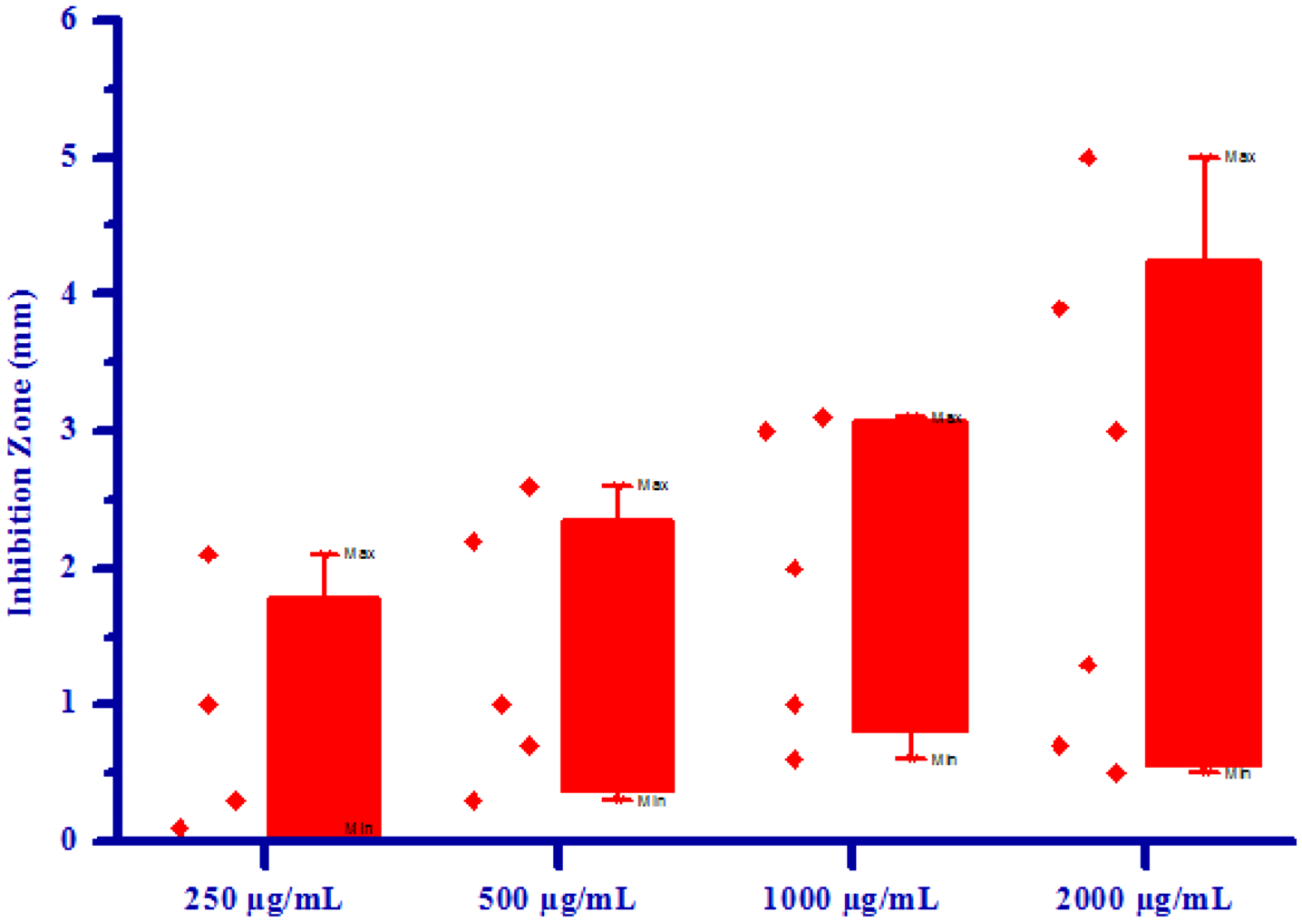
Box plot of substituted isoxazoles **(4a–4h)** against *Xanthomonas citri.*

**Figure 14:**
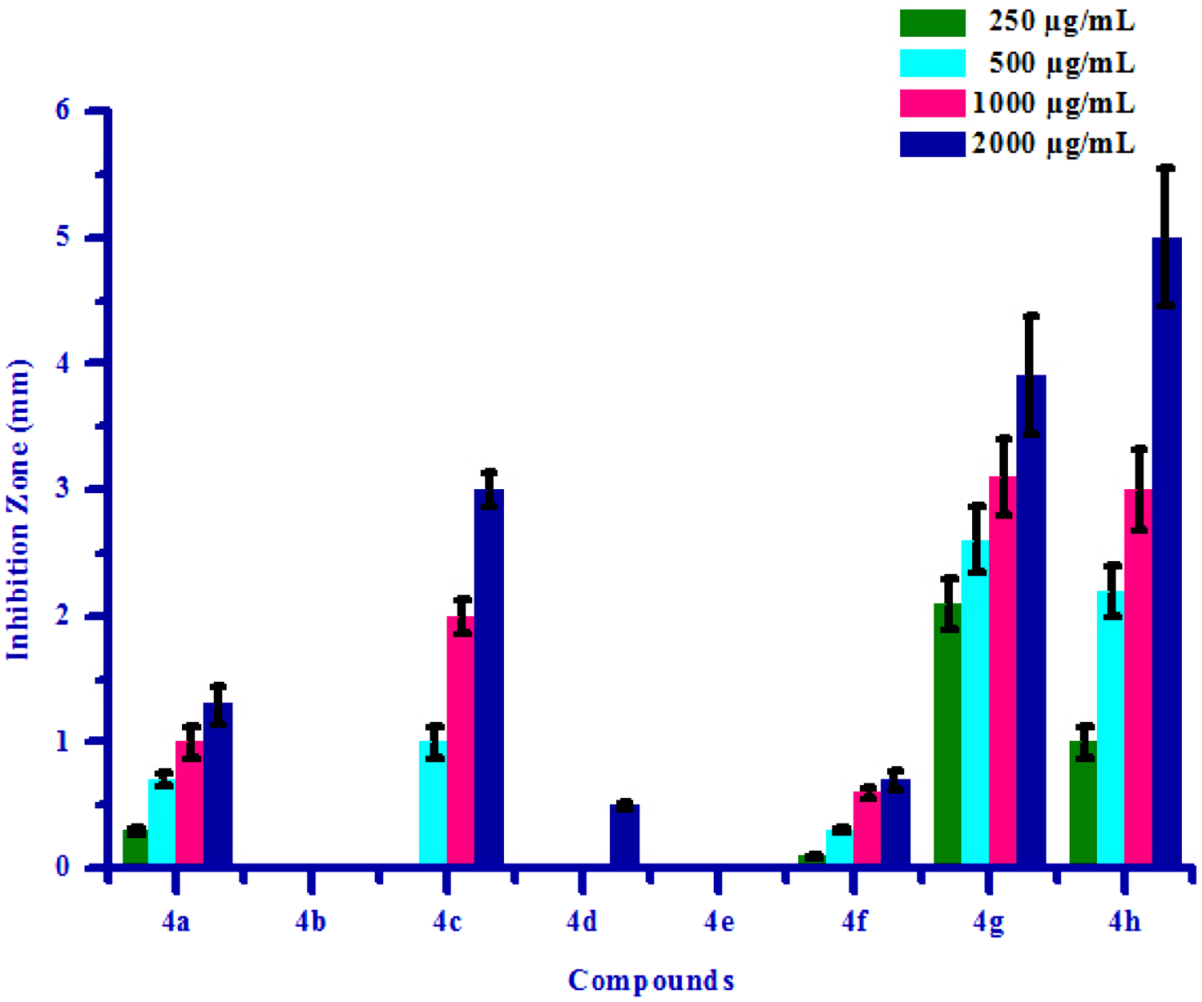
Antibacterial activity of substituted isoxazoles **(4a–4h)** against *Xanthomonas citri.*

The propitious antifungal activity of synthesized compounds **(4a–4h)** has inspired authors to test further for antibacterial activity. All synthesized compounds **(4a–4h)** were tested for their in vitro antibacterial activity against two bacterial strains *Erwinia carotovora* and *Xanthomonas citri* by inhibition zone method using DMSO as negative control. The results of antibacterial activity of synthesized compounds were shown in Table 6. Compound **4a** has shown no inhibition zone at 250 µg/mL concentration. Compound **4a** exhibited 1.00 mm, 2.00 mm and 3.00 mm inhibition zone against *Erwinia carotovora* at 500, 1000 and 2000 µg/mL concentrations respectively, due to electron withdrawing nature of chlorine group. Compound **4e** has shown no inhibition zone at all the concentrations against *Erwinia carotovora*, may be due to electron releasing nature of –OH group. Compound **4c** has shown no inhibition zone at 250 and 500 µg/mL concentrations respectively. Compound **4c** exhibited 2.00 mm and 5.00 mm inhibition zone at 1000 and 2000 µg/mL concentrations respectively, may be due to methoxy substitution on phenyl ring. Compound **4d** has shown no inhibition zone at 250 µg/mL concentration. Compound **4d** exhibited 1.00 mm, 2.50 mm and 4.00 mm inhibition zone against *Erwinia carotovora* at 500, 1000 and 2000 µg/mL concentrations respectively. Compound **4g** has shown no inhibition zone at 250 and 500 µg/mL concentrations respectively. Compound **4g** exhibited 0.60 mm and 1.20 mm inhibition zone against *Erwinia carotovora* at 1000 and 2000 µg/mL concentrations respectively. Compounds **4b, 4f** and **4h** exhibited 1.50, 2.20, 3.00, 4.00 mm, 0.70, 1.00, 1.60, 2.10 mm and 3.00, 5.50, 7.00, 9.60 inhibition zone against *Erwinia carotovora* at 250, 500, 1000 and 2000 µg/mL concentrations respectively, due to presence of methoxy and bromine groups on phenyl ring. Compounds **4b** and **4e** has shown no inhibition zone at all the concentrations against *Xanthomonas citri*. Compound **4d** has shown no inhibition zone at lower concentrations. Compound **4d** exhibited 0.50 mm inhibition zone against *Xanthomonas citri* at 2000 µg/mL concentration. Compound 4c has shown no inhibition zone at 250 µg/mL concentration. Compound **4c** exhibited 1.00 mm, 2.00 mm and 3.00 mm inhibition zone against *Xanthomonas citri* at 500, 1000 and 2000 µg/mL concentrations respectively, due to presence of methoxy group on phenyl ring. Compounds **4a**, **4f**, **4g** and **4h** exhibited 0.30, 0.70, 1.00, 1.30 mm, 0.10, 0.30, 0.60, 0.70 mm, 2.10, 2.60, 3.10, 3.90 mm and 1.00, 2.20, 3.00, 5.00 inhibition zone against *Xanthomonas citri* at 250, 500, 1000 and 2000 µg/mL concentrations respectively, mainly due to presence of chlorine, bromine and methoxy substitutions on phenyl ring. Maximum *Erwinia carotovora* growth was inhibited by compound **4h** showing inhibition zone 3.00–9.60 mm. Maximum *Xanthomonas citri* growth was also inhibited by compound **4h** showing inhibition zone 1.00–5.00 mm. This inhibition may be due to presence of methoxy group on phenyl ring. The box plot and graphical representation of antibacterial activity of all synthesized compounds **(4a–4h)** against *Erwinia carotovora* and *Xanthomonas citri* were shown in Figs. 11, 12, 13 and 14.

**Table 6:** Antibacterial activity of substituted isoxazoles **(4a–4h)**.

Compounds	Inhibition zone (mm)							
	Bacteria							
	*Erwinia carotovora* (conc.) µg/mL				*Xanthomonas citri* (conc.) µg/mL			
	250	500	1000	2000	250	500	1000	2000
**4a**	a	1.00 ± 0.05	2.00 ± 0.25	3.00 ± 0.45	0.30 ± 0.03	0.70 ± 0.05	1.00 ± 0.12	1.30 ± 0.15
**4b**	1.50 ± 0.20	2.20 ± 0.05	3.00 ± 0.15	4.00 ± 0.15	a	a	a	a
**4c**	a	a	2.00 ± 0.15	5.00 ± 0.45	a	1.00 ± 0.12	2.00 ± 0.13	3.00 ± 0.13
**4d**	a	1.00 ± 0.05	2.50 ± 0.25	4.00 ± 0.40	a	a	a	0.50 ± 0.02
**4e**	a	a	a	a	a	a	a	a
**4f**	0.70 ± 0.07	1.00 ± 0.01	1.60 ± 0.20	2.10 ± 0.25	0.10 ± 0.01	0.30 ± 0.02	0.60 ± 0.04	0.70 ± 0.07
**4g**	a	a	0.60 ± 0.07	1.20 ± 0.15	2.10 ± 0.20	2.60 ± 0.26	3.10 ± 0.30	3.90 ± 0.47
**4h**	3.00 ± 0.40	5.50 ± 0.05	7.00 ± 0.45	9.60 ± 0.36	1.00 ± 0.12	2.20 ± 0.20	3.00 ± 0.32	5.00 ± 0.55

All values are mean ± S.D.

a: no inhibition zone.

## Materials and methods

All the required chemicals for experiment were purchased from CDH (Central Drug House), SRL (Sisco Research Laboratory) and Sigma-Aldrich and used without purification. Melting points were determined in open head capillaries and are uncorrected. The reaction was monitored by thin layer chromatography. Infrared spectra (4000–350 cm^−1^) of the synthesized compounds were recorded in KBr pellets on Perkin Elmer FT-IR-R2X spectrophotometer and frequency was expressed in cm^−1^. The ^1^H NMR spectra were recorded in CDCl_3_ or DMSO-*d*_*6*_ using tetramethyl silane (TMS) as internal reference on “Brucker Ac 400 F” (400 MHz) nuclear magnetic resonance spectrometer. Elemental analysis was performed using ThermoFinnigan CHN elemental analyser. The chemical shifts values were quoted in delta (parts per million, ppm).

### Preparation of biocatalyst

#### Extraction of *Cocos nucifera* L. juice

The main ingredients per 100 g of coconut juice of *Cocos nucifera* are water (94.99 g), carbohydrates (3.71 g), protein (0.72 g), fat (0.2 g), ascorbic acid (2.4 mg) and pantothenic acid (0.043 mg). Coconut juice also contains many natural occurring bioactive enzymes such as acid phosphatase, catalase, dehydrogenase, diastase, peroxidase, RNA-polymerase etc. Due to presence of ascorbic acid and pantothenic acid, coconut juice is weakly acidic in nature. The coconut juice was obtained by holing the fruit with a knife. Then juice was filtered using Whatman filter paper no. 1 for removal of residues to get clear juice, which was used as catalyst^36^.

#### Preparation of *Solanum lycopersicum* L. juice

The main constituents per 100 g of *Solanum lycopersicum* L. juice are water (94.24 g), carbohydrates (3.53 g), protein (0.85 g), fat (0.29 g), ascorbic acid (70.1 mg), sugars (2.58 g) and dietary fibre (0.4 g). Fresh tomatoes were purchased from the local market. Then washed thoroughly under running tap water followed by rinsing thrice with double distilled water. Tomatoes were squeezed and juice were strained initially through a muslin cloth then passed through Whatman filter paper no. 1^37^.

#### Preparation of *Citrus limetta* juice

*Citrus limetta* is a species of citrus. It contains high amount of ascorbic acid due to which it acts as acid catalyst in organic synthesis. First of all wash the sweet limes and pat them dry. Cut them into two halves. Then using a citrus juice squeezer, juice was extracted. Then the juice was filtered through cotton and then through Whatman filter paper no. 1 to remove solid material and to get clear juice which was used as a catalyst.

### General procedure for the synthesis of substituted isoxazole derivatives (4a–4h)

#### By *Cocos nucifera* L. juice (method A)

A mixture of hydroxylamine hydrochloride (20 mmol) **(3a)**, methyl acetoacetate (20 mmol) **(2a)**, and *Cocos nucifera* L. juice (10 mL) in 20 mL water: ethanol (19:1) was stirred at room temperature for 15 min, then substituted aldehydes (20 mmol) **(1a–1h)** were added to mixture. The progress of reaction was monitored by thin layer chromatography (Scheme 1). The solid was separated out, then filtered and washed with ice cold water to get the products **(4a–4h)**, which was further recrystallized with methanol. All synthesized compounds **(4a–4h)** were characterized by ^1^H NMR, FTIR and CHN analyses.

#### By *Solanum lycopersicum* L. juice (method B)

A mixture of hydroxylamine hydrochloride (20 mmol) **(3a)**, methyl acetoacetate (20 mmol) **(2a)**, and *Solanum lycopersicum* L. juice (10 mL) in 20 mL water: ethanol (19:1) was stirred at room temperature for 15 min, then substituted aldehydes (20 mmol) **(1a–1h)** were added to mixture. The progress of reaction was monitored by thin layer chromatography (Scheme 1). The solid was separated out, then filtered and washed with ice cold water to get the products **(4a–4h)**, which was further recrystallized with methanol. All synthesized compounds **(4a–4h)** were characterized by ^1^H NMR, FTIR and CHN analyses.

#### By *Citrus limetta* juice (method C)

A mixture of hydroxylamine hydrochloride (20 mmol) **(3a)**, methyl acetoacetate (20 mmol) **(2a)**, and *Citrus limetta* juice (10 mL) in 20 mL water: ethanol (19:1) was stirred at room temperature for 15 min, then substituted aldehydes (20 mmol) **(1a–1h)** were added to mixture. The progress of reaction was monitored by thin layer chromatography (Scheme 1). The solid was separated out, then filtered and washed with ice cold water to get the products **(4a–4h)**, which was further recrystallized with methanol. All synthesized compounds **(4a–4h)** were characterized by ^1^H NMR, FTIR and CHN analyses.

### Screening of herbicidal activity

Solutions of 50 µg/mL, 100 µg/mL, 150 µg/mL and 200 µg/mL of the test compounds in DMSO were prepared. Agar powder (5gm) was put into boiling distilled water (1L) until it dissolved, and then cooled down to 40–50 °C. The solution (2 mL) containing test compounds and melting agar (18 mL) was mixed and this mixture was added to a petridish with 4.5 cm diameter. The agar plate without test compound was used as an untreated control. The 15 seeds of *Raphanus sativus* L*.* (Radish) were put on the surface of the agar plate. The Petridishes were covered with glass lids, and the cultivation conditions were kept at 25 ± 1 °C and 12 h in light and 12 h in dark alternating for seven days. Seven days later, the root lengths and shoot lengths of *Raphanus sativus* L. were measured. The growth inhibitory rate related to untreated control was determined by given formula.

$$\mathrm{\% Inhibition }= \frac{\mathrm{Control}-\mathrm{Treated }}{\mathrm{Control}}\times 100.$$

### Screening of antifungal activity

Amongst the several methods available, poisoned food technique^38^ which is the most common was used for testing antifungal activity. The test fungus was grown on Potato dextrose agar medium. The required amount of synthesized compounds dissolved in 1 mL of DMSO was incorporated aseptically into 99 mL aliquots of sterilized potato dextrose agar cooled at 45 °C after brief shaking. Each lot of medium was poured into Petri dishes and allowed to solidify. 1 mL DMSO in media was taken as control. Each dish was inoculated centrally with a 5 mm mycelial disc cut from the periphery of 2–3 days old fungal colonies. Inoculated Petri plates were incubated in the dark 25 ± 2 °C for 48–72 h and colony diameters were measured periodically till the control dishes were nearly completely covered with fungus growth. Three replicates were used for each concentration of a chemical together with three dishes containing only the solvent and no toxicant. The degree of inhibition of growth was calculated from the mean differences between treatments and the control as percentage of latter by using the formula.

$$\mathrm{\% Inhibition }= \frac{\mathrm{Control}-\mathrm{Treated }}{\mathrm{Control}}\times 100,$$

where Control = mycelial growth in control dish, Treated = mycelial growth in treated dish.

### Screening of antibacterial activity

The inhibition zone method^39^ was followed for screening the synthesized compounds for their antibacterial activity. The bacterial suspension was prepared from 48 h old culture. The bacterial growth from five slants was taken and mixed in 100 mL sterilized distilled water aseptically. The medium was melted and cooled at 45 °C, needed medium was poured aseptically in sterilized Petri plates and rotated gently for even distribution of the medium and was allowed to solidify. 250, 500, 1000 and 2000 µg/mL concentrations of synthesized compounds were prepared from the stock solution by taking appropriate amount and diluting with DMSO. The circular paper discs of 10 mm diameter were prepared from Whatman’s Filter paper No. 1. The disc were kept in Petri plate and autoclaved at 15 lbs pressure 20 min. Two paper discs were used for each concentration of the synthesized compounds. The excess of solution absorbed by paper discs was removed by holding them vertically by sterile forecep. Such soaked discs were transferred aseptically to Petri plates containing media and bacterial suspension spread over the surface. Each concentration and chemical was replicated 3 times. Such Petri plates were inverted and kept at 5 °C for 2 h for better diffusion of the chemicals in agar medium. Later, on the Petri plates were incubated at 25 ± 2 °C for 48 h. The zone of inhibition for each concentration of the chemicals was recorded in mm after 48 h of incubation.

### Characterization data of selected compounds

#### (Z)-4-(3,4-dimethoxybenzylidene)-3-methylisoxazol-5(4H)-one (4b)

*Yellowish Solid*; m.p. 210–212 °C; ^1^H NMR (400 MHz, DMSO-*d*_*6*_): *δ* 2.30 (s, 3H, CH_3_); 3.87 (s, 3H, OCH_3_); 3.93 (s, 3H, OCH_3_); 7.87 (s, 1H, =CH); 7.10–8.47 (m, 4H, Ar–H); IR (*ν*_*max*_ cm^−1^) (neat): 1587.9 (C=N), 1610.0 (C=C, aromatic), 1742.1 (C=O); 1441.5 (N–O), 1284.6 (OCH_3_); Elemental Analysis found for (C_13_H_13_NO_4_): C (63.15, 62.89), H (5.30, 5.32), N (5.67, 5.78).

#### (Z)-4-(4-methoxybenzylidene)-3-methylisoxazol-5(4H)-one (4c)

*Yellowish Solid*; m.p. 172–174 °C; ^1^H NMR (400 MHz, DMSO-*d*_*6*_): *δ* 2.25 (s, 3H, CH_3_); 3.89 (s, 3H, OCH_3_); 7.74 (s, 1H, =CH); 7.05–7.07 (m, 2H, Ar–H); 8.47–8.50 (m, 2H, Ar–H); IR (*ν*_*max*_ cm^−1^) (neat): 1591.5 (C=N), 1619.0 (C=C, aromatic), 1729.9 (C=O); 1431.8 (N–O), 1276.6 (OCH_3_); Elemental Analysis found for(C_12_H_11_NO_3_): C (66.35, 66.33), H (5.10, 5.13), N (6.45, 6.62).

#### *(Z)-*4-(2-hydroxybenzylidene)-3-methylisoxazol-5(4H)-one (4e)

*Red Solid*; m.p. 198–200 °C; ^1^H NMR (400 MHz, DMSO-*d*_6_): *δ* 2.25 (s, 3H, CH_3_); 8.20 (s, 1H, =CH); 6.87–8.77 (m, 4H, Ar–H); 10.85 (s, 1H, OH); IR (*ν*_*max*_ cm^−1^) (neat): 1598.1 (C=N), 1631.5 (C=C, aromatic), 1736.8 (C=O); 1439.7 (N–O), 1285.9 (OCH_3_).

#### (Z)-4-(4-bromobenzylidene)-3-methylisoxazol-5(4H)-one (4f)

*Yellowish Solid*; m.p. 178–180 °C; ^1^H NMR (400 MHz, CDCl_3_): *δ* 2.29 (s, 3H, CH_3_); 7.37 (s, 1H, = CH); 7.59–8.22 (m, 4H, Ar–H); IR (ν_max_ cm^−1^) (neat): 1593.5 (C=N), 1616.5 (C=C, aromatic), 1725.7 (C=O); 1430.2 (N–O), 1277.5 (OCH_3_).

### Statistical analysis

The experiments were performed in triplicates for each treatment and the mean value were recorded and expressed as mean ± S.D. The descriptive statistics in form of box-and-whisker diagram were also presented in this paper. The spacing between the different parts of the box indicates the degree of dispersion and skewness in the data (Supplementary Fig. S1).

## Conclusions

We have developed a novel route for synthesis of biologically active substituted isoxazole derivatives **(4a–4h) **via one-pot three-component reaction between substituted aldehydes **(1a–1h),** methyl acetoacetate **(2a)** and hydroxylamine hydrochloride **(3a)** in presence of *Cocos nucifera* L. juice, *Solanum lycopersicum* L. juice and *Citrus limetta* juice. The present protocol offers many advantages such as simple and efficient catalytic system, simple work-up, cost-effective and products were obtained in good to excellent yields. A comparison between current catalysts viz. *Cocos nucifera* L. juice, *Solanum lycopersicum* L. juice & *Citrus limetta* juice and some previous catalysts for synthesis of substituted isoxazole derivatives revealing that these catalysts are superior to other reported catalysts in terms of product yield, reaction time and catalyst loading. All synthesized compounds **(4a–4h)** were also screened for their bio efficacy in terms of herbicidal activity against *Raphanus sativus* L*.* (Radish) seeds, fungicidal activity against *R. solani*, C. *gloeosporioides* and antibacterial activity against *Erwinia carotovora* and *Xanthomonas citri*. Based on biological activity data, we concluded that strong electronegative groups at the phenyl ring exhibit a good activity profile compared to electron releasing groups. This research work also encourage organic chemist for the design of novel molecules to identify many more biologically active heterocycles for the benefit of humanity.

## Supplementary Information

Supplementary Figures.
