# Supplementary material for: Fruit juice mediated multicomponent reaction for the synthesis of substituted isoxazoles and their in vitro bio-evaluation
Source: Sci Rep. 2021 Dec 7;11:23563. doi: 10.1038/s41598-021-03057-6 (PMC8651685; doi:10.1038/s41598-021-03057-6)
Supplement: Supplementary file 1 — Supplementary Figures. [file 41598_2021_3057_MOESM1_ESM.docx]

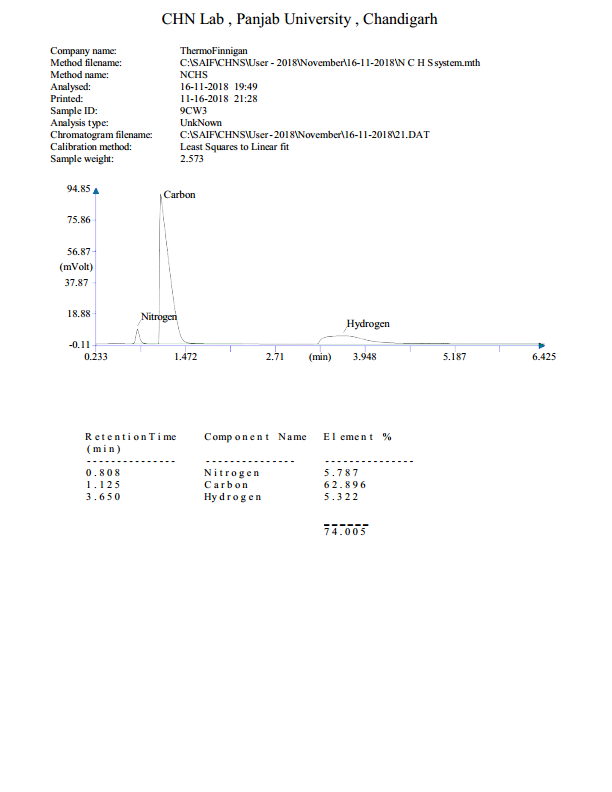
 **S1 Fig. CHN analysis of compound (4b)**

**
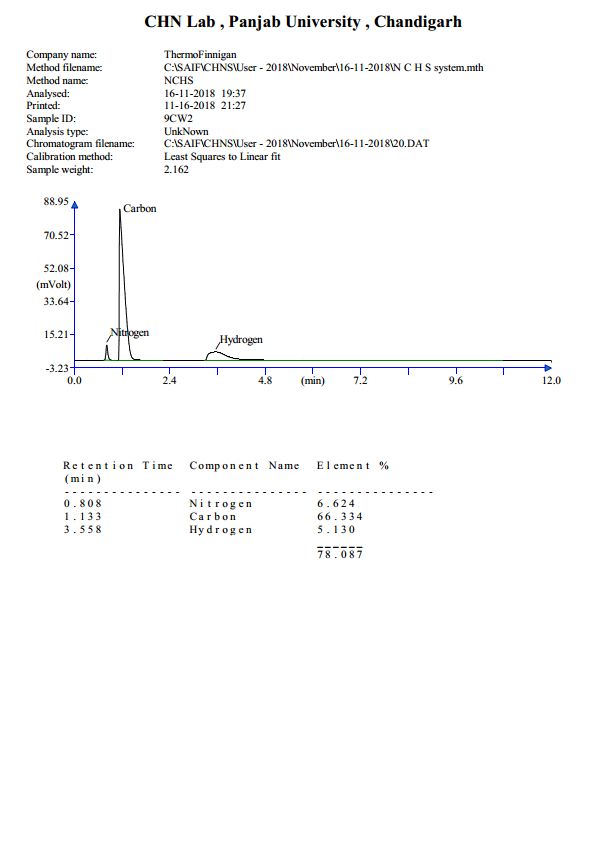
**

**S2 Fig. CHN analysis of compound (4c)**


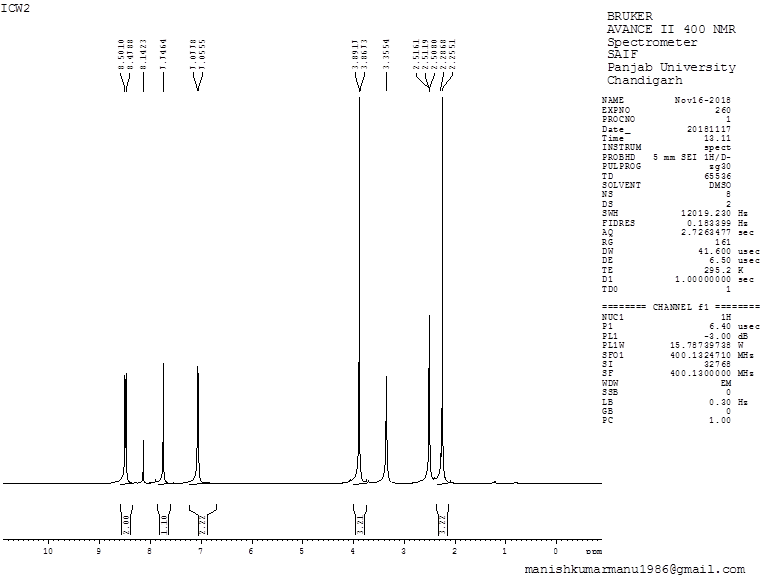


**S3 Fig. 1HNMR spectrum of compound (4c)**


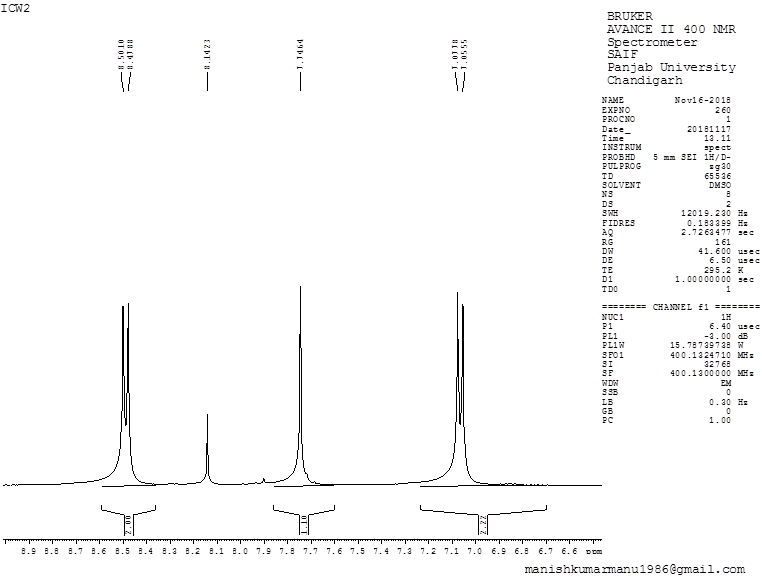


**S4 Fig. 1HNMR spectrum of compound (4c) (Expanded form 1)**

**
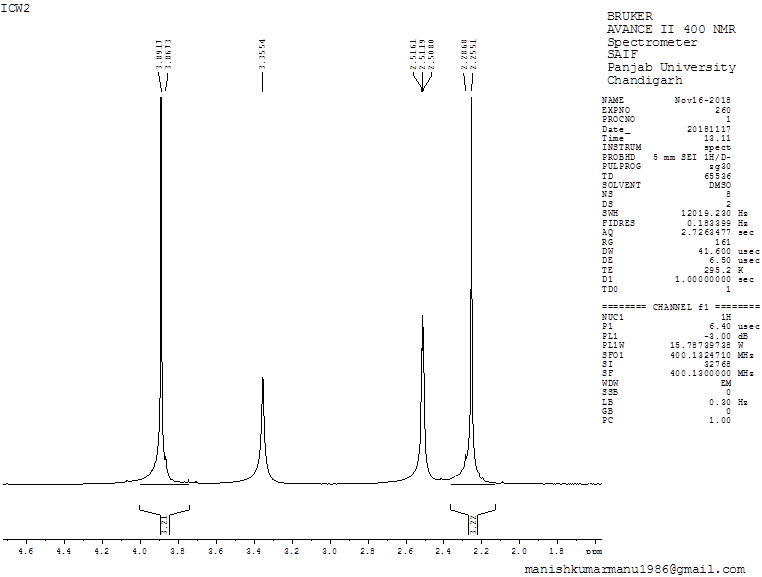
**

**S5 Fig. 1HNMR spectrum of compound (4c) (Expanded form 2)**


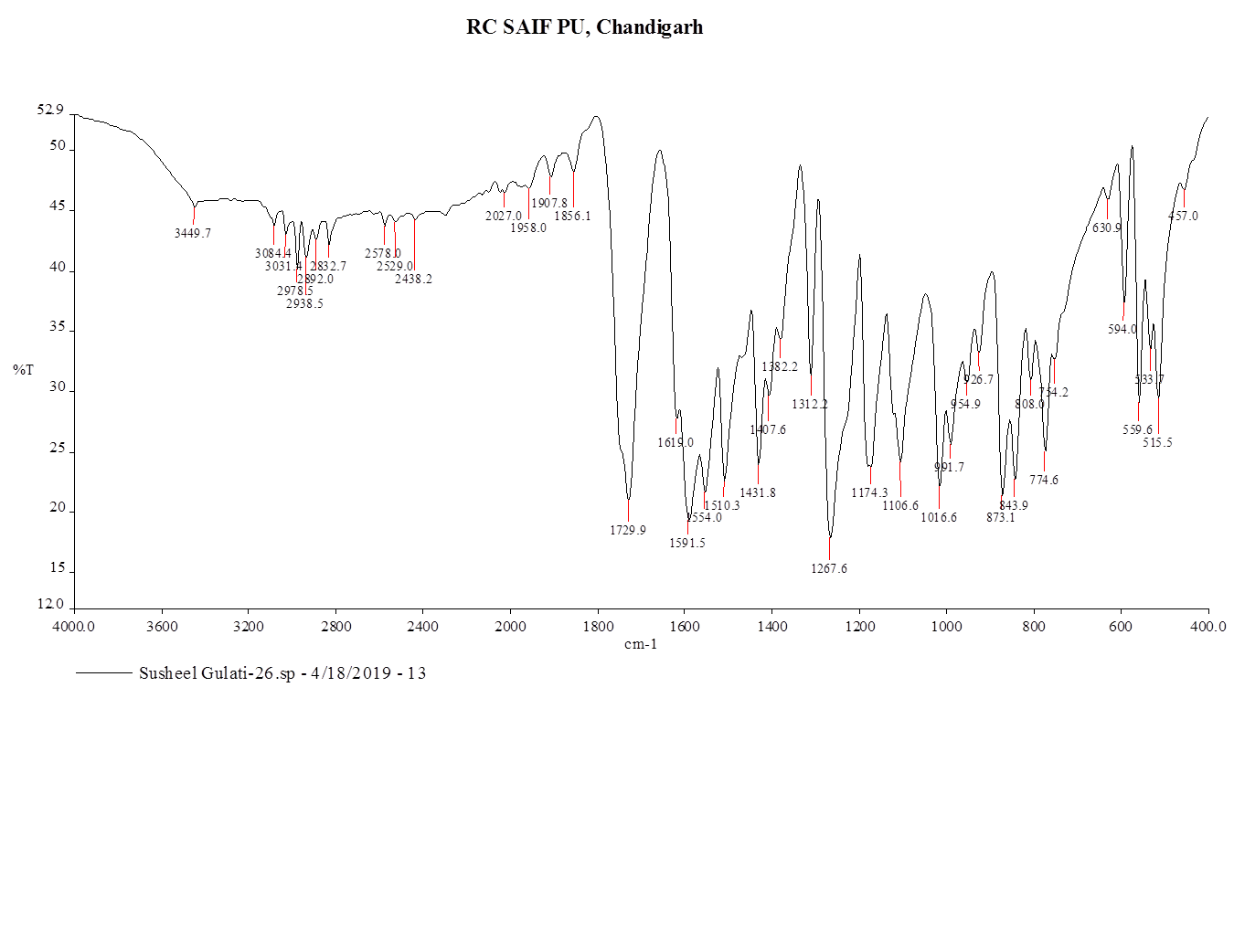


**S6 Fig. FTIR spectrum of compound (4c)**


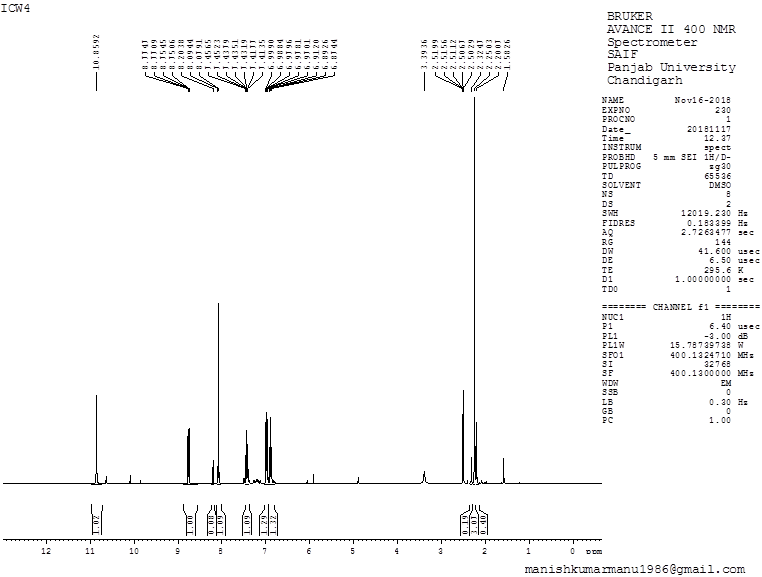


**S7 Fig. 1HNMR spectrum of compound (4e)**


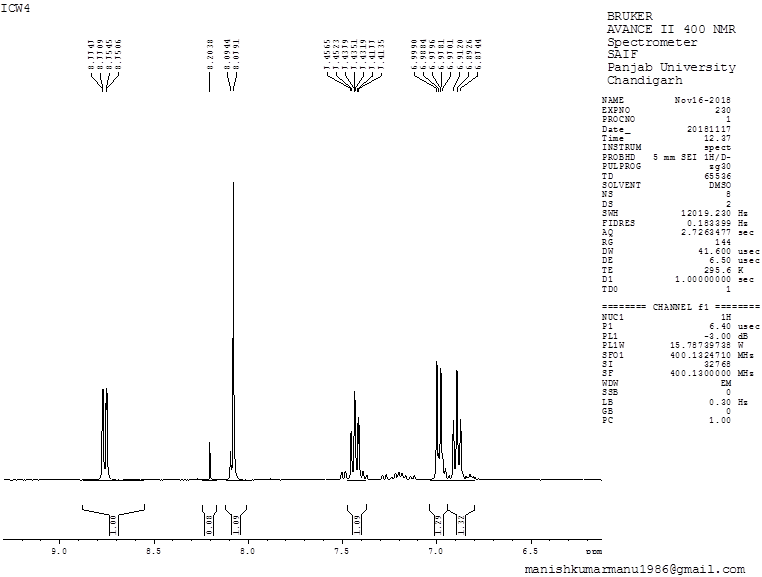


**S8 Fig. 1HNMR spectrum of compound (4e) (Expanded form 1)**


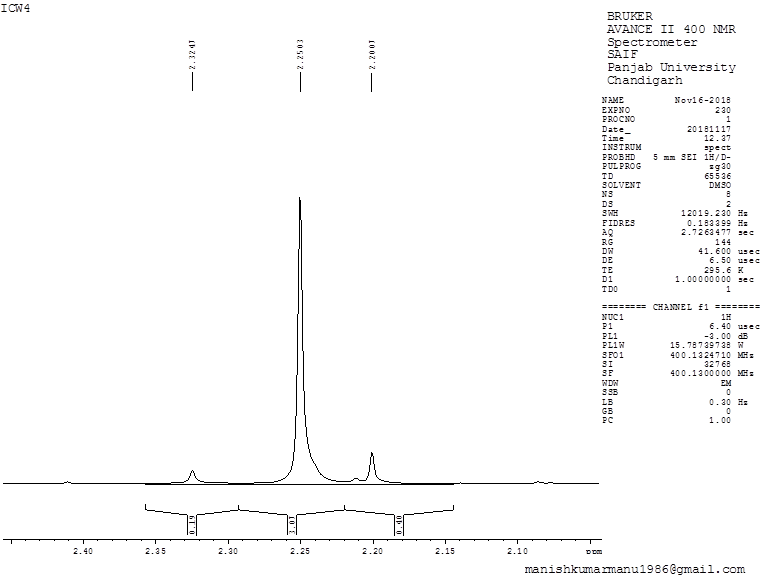


**S9 Fig. 1HNMR spectrum of compound (4e) (Expanded form 2)**


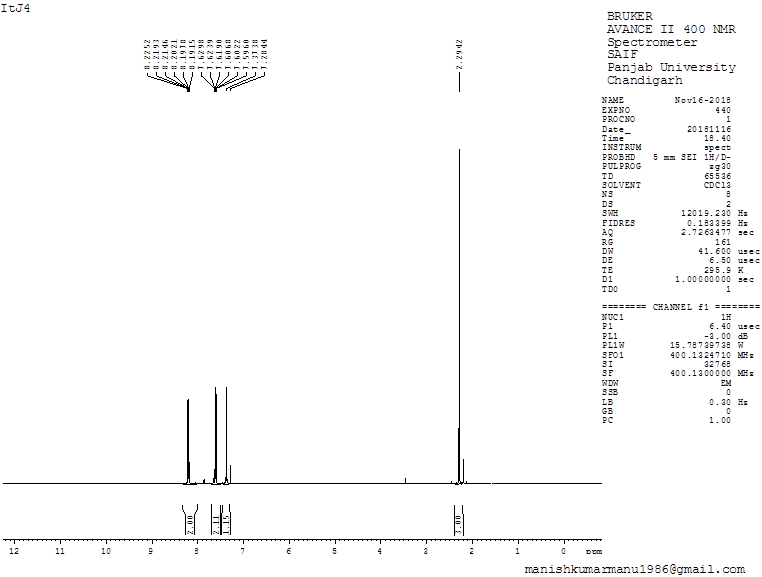


**S10 Fig. 1HNMR spectrum of compound (4f)**


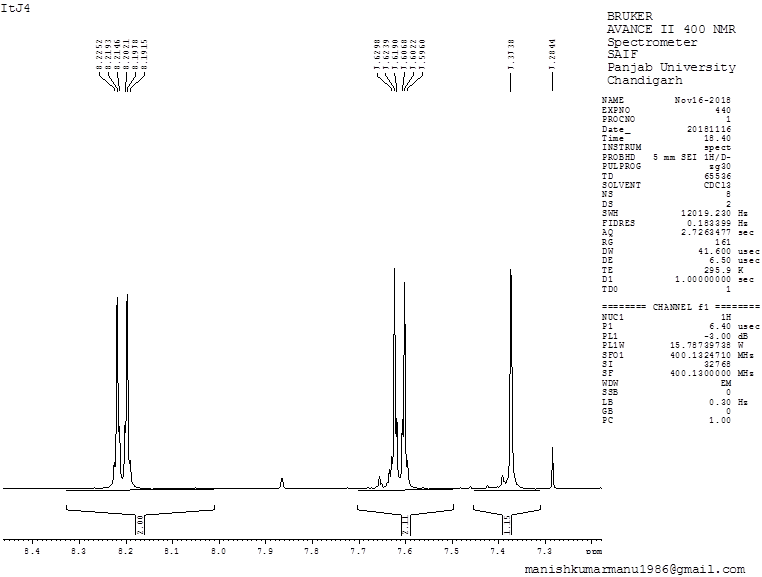


**S11 Fig. 1HNMR spectrum of compound (4f) (Expanded form 2)**
